# Externalities of Lean Implementation in Medical Laboratories. Process Optimization vs. Adaptation and Flexibility for the Future

**DOI:** 10.3390/ijerph182312309

**Published:** 2021-11-23

**Authors:** Simona Andreea Apostu, Valentina Vasile, Cristina Veres

**Affiliations:** 1 Department of Statistics and Econometric, Faculty of Cybernetics, Statistics and Economic Informatics, Bucharest University of Economic Studies, 010552 Bucharest, Romania; 2 Institute of National Economy-Romanian Academy, 050711 Bucharest, Romania; valentinavasile2009@gmail.com; 3 Industrial Engineering and Management Department, Faculty of Engineering and Information Technology, George Emil Palade University of Medicine, Pharmacy, Science, and Technology of Targu Mures, 540142 Targu Mures, Romania; cristina.veres@umfst.ro

**Keywords:** Lean Six Sigma, healthcare, clinical laboratory, bibliometric analysis, regression analysis

## Abstract

Important in testing services in medical laboratories is the creation of a flexible balance between quality-response time and minimizing the cost of the service. Beyond the different Lean methods implemented so far in the medical sector, each company can adapt the model according to its needs, each company has its own specifics and organizational culture, and Lean implementation will have a unique approach. Therefore, this paper aims to identify the concerns of specialists and laboratory medical services sector initiatives in optimizing medical services by implementing the Lean Six Sigma method in its various variants: a comparative analysis of the implemented models, with emphasis on measuring externalities and delimiting trends in reforming/modernizing the method, a comprehensive approach to the impact of this method implementation, and an analysis of available databases in order to underline the deficit and information asymmetry. The results highlighted that in the case of clinical laboratories, the Lean Six Sigma method is conducive to a reduction of cases of diagnostic errors and saves time but also faces challenges and employees’ resistance in implementation.

## 1. Introduction

The diffusion of digitalization in medicine is dynamic and includes the entire chain of medical services, from analyzes and tests for diagnosis to postoperative and post-recovery evaluations of patients. In diagnosis, the time and quality of test results represent the cornerstone for the subsequent evolution of the process of diagnosis, intervention, treatment, and recovery, from the perspective of time and the quality and efficiency of the medical treatment decision. In this context, the automation of testing services is an imminent development and a step towards increasing managerial efficiency, with an impact on reducing the response time for the patient. Equally important is the interpretation of test processing by the specialist doctor in the laboratory, specifically professional expertise and work experience being essential. In this process of interpreting the results and diagnosis, the help provided by historical databases and, more recently, AI (Artificial Intelligence) becomes necessary, as an accompanist/facilitator in the specialist’s laboratory work, especially on the sequence of the final decision—the test result. AI assistance and big data normally influence employment profiles and/or upgrade digital skills and determine jobs destruction [1] and labor productivity increase [2] but with different incidents on fields of activity. Therefore, in testing laboratories in the medical sector, the most important effect is the need to update digital skills to facilitate the digitization of processing and interpretation of results, assisted by AI and big data. Increases regarding the number of tests per unit of time, but also the quality of services through a high potential to avoid diagnostic errors, AI acts as comparative support for similar tests, a “second opinion,” but resulting from querying historical databases with test results and associated diagnosis.

The Lean Six Sigma (LSS) method is a continuous improvement methodology that combines Lean Philosophy and Six Sigma methodology [3], aimed to increase an institution’s or company’s profit and development by making their process more efficacious [4]. Lean is recognized for its ability to handle waste and Six Sigma is known for process improvement [5,6].

LSS has become the most popular business strategy for deploying continuous improvement in the manufacturing and service sectors as well as in the public sector [7,8].

The interest in Lean in the medical field has expanded in the last decades. The continuous growth of the global population, correlated with the increase in life expectancy and the significant improvements of diagnosis processes and treatment approaches has led to raised demands and high expectations in terms of costs and quality services in healthcare systems worldwide, including laboratory tests.

Special attention is placed in this article on LSS application in clinical laboratory environments. This is not by chance: 60–70% of patient management decisions are heavily reliant on clinical laboratory results, and innovations in this field that maximize productivity yet reduce costs are highly warranted [9].

Applied in its various forms, LSS demonstrates that the optimization of laboratory activities associated with processing based on modern equipment and technologies complements the factors that determine the quality of the diagnosis and, potentially, minimizes/reduces interpretation/diagnostic errors. It is also the reason why laboratories apply such a managerial method. In practice, the application of Lean allows to “improve quality, patient safety, and workflow in histology and anatomic pathology” [10]. It is in fact an improvement of the entire laboratory process, allowing quality improvement process on efficiency and quality [11], with minimal errors [12]. Finally, Lean helps in “changing the mindset” of an organization toward providing the highest quality of services with faster delivery at an optimal cost [13]. It is worth mentioning that the efficiency of the activity and the reduction of costs will allow laboratories not only to optimize staff allocation and increase the number of tests but will also facilitate flexibility in adapting the business to digital pathology with a remote diagnostic histopathology service [14], reducing the total time of the service and, additionally, decreasing the transport of the slats.

LSS represents the most effective technique in process improvement, being widely implemented in top-performing organizations. Although its origin is in the manufacturing sector, it is also suitable for services [15]. It is described as a well-structured theory-based methodology with the aim to improve performance and develop effective leadership, customer satisfaction, and, at the bottom line, results [16]. Together, lean manufacturing and six sigma become more powerful, eliminating the cons of each approach, as it applies both OLS and techniques [17].

In this sense, the main aim of our paper is to analyze the application of Lean Six Sigma in clinical laboratories, highlighting its advantages. To meet this objective, we envisaged four research questions. (i) What are the most common terms regarding Lean Six Sigma methods? (ii) How are clinical laboratories distributed around the world? (iii) Is there a link between hospitals, laboratories, inpatient facilities, and outpatient facilities? (iv) Is there an inter-determination between the number of clinical laboratories and the number of hospitals, explaining health status? Our research relies on two types of data: in order to identify the terms related to Lean Six Sigma, we used 1661 articles provided by Web of Science and 720 abstracts provided by PubMed (bibliometric analysis) and, in order to investigate the application of Lean Six Sigma methods on clinical laboratories, we used the data provided by the World Health Organization (WHO) on Global Antimicrobial Resistance and Use Surveillance System (GLASS) Report: 2021.

The novelty of the manuscript is represented by a bibliometric analysis of content, focused on the application of lean in medical laboratories, a critical state analysis of existing statistical information and recommendations, and the delimitation of the coordinates of the laboratory testing medical services model, organized according to the lean, digitized principles, with the incorporation of AI as a “working” tool.

The originality of our research is given by the integrated approach, as it incorporates both qualitative and quantitative analysis. The qualitative analysis is realized at the level of a literature review in order to find the most common words associated with the Lean method, and the quantitative analysis highlights new evidence about the dependency between the number of hospitals and the number of clinical laboratories and in- and outpatient facilities.

Therefore, the paper is structured as follows: the literature review section offers an overview of the most relevant studies regarding the topics of interest that are investigated in our research, Section 3 outlines a theoretical background of the methods used in the quantitative analysis, Section 4 presents the bibliometric analysis results, and Section 5 details the empirical results whereas the last section presents discussions and the main conclusions of the research.

## 2. Literature Review

The interest in Lean in the medical field has expanded in the last decades. The increase of the global population, correlated with the increase in life expectancy and the progress of diagnosis and treatment, has led to raised demands and high expectations in terms of the cost and quality of services in healthcare systems worldwide.

Several large European hospitals have embraced the Lean Six Sigma application to improve workflow processes related to healthcare, with benefits in increasing the quality of patient care as well as lowering costs [18].

Clinical laboratories are healthcare facilities providing a wide range of laboratory procedures that aid physicians in carrying out the diagnosis, treatment, and management of patients [19]. Clinical laboratories play an important role in diagnosis. They are expected to promptly deliver highly reliable data and results and give accurate input for timely disease diagnosis, prevention, and health control. 

Besides this, clinical laboratories must handle increasing workloads with a broader spectrum of parameters with the same (or fewer) amount of staff and must still deliver consistent results with improved turnaround times (TATs) and with utmost quality [20].

As quality management systems (QMS) for laboratories are the sound ground for the management of quality in health laboratories [21], Lean Six Sigma methodology can contribute substantially in achieving, maintaining, and improving the accuracy, timeliness, and reliability of results of clinical laboratories.

We selected the articles from Web of Science that relate to the Lean Six Sigma application in clinical laboratories. We were interested in summarizing the positive and negative effects, the methodology of implementation, if there is any described, as well as the Lean methods mentioned in recent research papers, considering only those published from 2018 so far. Review articles were excluded from the study, as they refer to the implementation of Lean Six Sigma methodology before 2018. 116 articles were found on the Lean Six Sigma implementation in a laboratory, by searching for the keywords Lean Six Sigma and Laboratory. Only 36 of them were written in the period 2018–2021.

Each article was studied in order to identify those which relate to healthcare. References of the relevant articles were also studied to identify valuable studies to be added to our analysis. Content research was conducted so that the table presents only actual, relevant, and valuable in the context studies.

There are two categories of Lean Six Sigma applications in clinical laboratories:

(1) Application of the Six Sigma breakthrough methodology to solve problems, reduce defects, and better satisfy customers;
(2) Quantification of laboratory test performance on the Sigma scale [22].

Of the studies, we identified and selected only articles in the first category. 

Table 1 contains an overview of the recent Web of Science articles that present the LSS application in a clinical laboratory environment, presenting the area and place of implementation, method, and mentioned instruments, effects, and challenges.

Unique interesting elements were identified in almost each research paper. Durur and Akbulut [23] highlighted key elements in order to achieve success in lean methodology implementation: the continuous training of employees and their participation in the process.

The financial part is debatable. In their work, Isack et al. [24] listed enablers and barriers of Lean principles. Staff resistance to change is one of the main barriers, along with lack of support from management and financial constraints. On the opposite side, Letelier [25] et al. mentioned that results were achieved with minimum financial investment (except for the structural modifications). Another case study in a tertiary care pediatric hospital in Nova Scotia, Canada, did not require high financial or time investment, as mentioned by Geerlinks et al. [31].

As Kovach et al. [26] highlighted in their study, a nonprofit health care clinic in Texas has previously worked on implementing process improvement, and their own resourcefulness was quickly being exhausted, so the LSS approach was needed to achieve further performance.

Compared to others who used the DMAIC cycle (Define–Measure–Analyze–Improve–Control), Moran et al. [27] used DMADV (Define–Measure–Analyze–Design–Verify), pointing out that the last phase (Verify) ensures a control plan is incorporated into the quality improvement project as a sustainable protocol.

Ahmed et al. [28] from Malaysia discovered in one of their studies that Six Sigma methodology develops a unique competency, difficult to duplicate by competitors. The concept of Six Sigma initiatives is not static but dynamic in nature; while deepening and extending their research that year [29] they found that senior management commitment has no direct significant relationship with quality performance but has an indirect significant relationship with quality performance through the mediating effects of LSS and workforce management.

Persis et al. [30] presented in their research the implementation of a more extensive approach SDMMAICS (Select–Define–Measure–Map–Analyze–Improve–Control–Sustain) with detailed steps. The article highlights an annual saving of USD 44,000, while the implementation was done with a team of Black Belt LSS-certified industrial experts, which ensured project sustainability. The alternative model was proposed by two of the authors of the article in 2018.

Other advantages of LSS implementation in clinical laboratory environments are presented in the literature: the optimization of flows, ergonomics, and organization of workstations, creation of a more pleasant environment [34], reduction in delays, some budgetary gains, and improvement in the satisfaction of involved personnel [35].

The research showed the most recent implementations of Lean Six Sigma in clinical laboratory environments, pointing out the current improvements, used instruments, and effects. We were interested in the Lean Six Sigma application specifically in medical laboratories studies with a specific characteristic—those who describe implementation as a management approach.

We remarked that articles are usually written from the organizer’s perspective, without presenting any employees’ perspective feedback, which usually has a different overview of the improvements than the owners. 

Letelier et al. [25] mentioned that implementation contributed to the emergence of a culture of continuous improvement in the laboratory. Still, analyzing all the selected articles, we can conclude that too little emphasis was placed on the effects that Lean has on organizational culture and on improving relationships within work teams and focused only on quantitative performance measurement, specifically on the analysis of key performance indicators. Lean implementation means a paradigm shift in laboratory efficiency and an alignment of principles and values that should, in theory, strengthen the work team, improve productivity and work quality, and reduce the share of erroneous tasks. People are the ones who generate change in organizations; however, we noticed that there is a lack of concern/analysis on the reaction/resistance of employees to change, and conflicts may arise during the implementation of new managerial and organizational tools such as LSS. Training is an essential step in Lean Six Sigma implementation in any organization. By exception, we identified one study where, in the process of implementation, specifically during the training session with laboratory staff to educate them about the benefits of Lean implementation, the need of their contribution, and the importance of their work for overall system efficiency and patient satisfaction was highlighted. The training was also aimed at assuring them that the process change brought in Lean implementation would neither threaten their jobs nor would be a measure of their efficiency. They were advised to work as per their regular pace and were encouraged to give their feedback for improvements [13]. To our vision, this is an important ingredient for the successful implementation of LSS and for preventing people’s resistance to change and conflicts. Information sessions and courses for all employees are needed for a clear understanding of the scope and for obtaining adequate involvement, highlighting that LSS aims to improve the process and not reduce staff. In a laboratory environment, both doctors and operators should understand the fact that efforts towards adapting the method do not lead to a diminishing of the specialist’s importance but help them consolidate accurate diagnosis, reduce errors, increase laboratory capacity, provide better quality and a quicker result and, finally, redesign work content according to organizational dynamics. Clinical laboratories are mandated to deliver accurate, reliable, timely, and correctly reported results which are used in decision making for disease screening, diagnosis, and monitoring [36].

According to MarketResearch.com [37] the clinical laboratory services market will grow annually by 5.9% during the 2020–2027 period. Considering the large number of existing laboratories worldwide, we consider that the concern towards high-performance managerial tools such as LSS implementation is still very limited, despite good practice examples.

The analysis of the selected articles shows low interest in the identification and analysis of negative externalities in the LSS implementation process. As we can notice in the presented table, there are very few articles that present a subjective account of the negative effects or challenges because of LSS implementation. Authors usually focus on positive achievements as a “halo” effect, but, as research shows, there are negative consequences as well. The resistance of employees, conflicts between management and other decision levels which may arise, giving up or additional changes before achieving the expected results and impact assessment, improving key performance indicators values to the detriment of service quality, time investment, and other issues must be known and assumed by all employees and relevant stakeholders before starting LSS implementation. Many work teams push to turn back to the old way of working after the improvement projects are over or/and after the external specialized implementation team leaves. Studying the challenges of LSS implementation may be a good direction for valuable research. The analysis of LEAN implementation and process optimization is not enough; feedback is needed in the post-implementation period to determine the medium/long-term effects from the perspective of all those involved. 

Some studies [38] conclude that implemented lean tools do not eliminate particulate waste, as it was assumed at the initial stage, and that the plan of implementation of particular tools was also not completely applied in practice. Further analysis is needed to quantify effects on a long-term basis.

Based on previous theoretical considerations, the following hypotheses have been created in order to stipulate the applicability of Lean Six Sigma methods on clinical laboratories:

**Hypothesis** **1** **(H1):** *The most common terms regarding Lean Six Sigma methods imply quality, patient, and hospital*.

**Hypothesis** **2** **(H2):** *Clinical laboratories are distributed differently around the world depending on the level of development*.

**Hypothesis** **3** **(H3):** *There is a link between hospitals, laboratories, inpatient facilities, and outpatient facilities, regardless of geographical area*.

**Hypothesis** **4** **(H4):** *There is an interdetermination between the number of clinical laboratories and the number of hospitals, explaining health status*.

## 3. Data and Methodology

In order to analyze the most common words regarding Lean Six Sigma methods, we used a bibliometric analysis. Bibliometric analysis investigates literature in a systemic and systematic process, structuring and ordering the results obtained converted from quantitative to qualitative.

Bibliometric methods provide quantitative analysis in the case of written publications, being related to the terms “infometrics” [39,40] and “scientometrics” [41].

This analysis involves the identification of the literature’s content, i.e., within a given subject area. Therefore, the scientific production is evaluated, the results being of major importance to policymakers, scientists, or other stakeholders [42]. Bibliometric analysis is considered a state-of-the-art methodology, including components from all scientific domains [43].

In order to identify the main topic of the content, we used word clouds, considering the words with the highest frequency. The relationships between words can be determined by investigating which words tend to immediately follow others or that tend to co-occur within the same documents. Both types of analyses are complementary. If the word network reveals which word pairs co-occur most often, the correlation network reveals which words appear more often.

In order to investigate the application of Lean Six Sigma methods in clinical laboratories, we used data provided by the World Health Organization (WHO) on Global Antimicrobial Resistance and Use Surveillance System (GLASS) Report: 2021 [44]. The data indicated the number of local clinical laboratories and number of surveillance (hospitals, laboratories, and in- and outpatient facilities) in Africa, America, the Eastern Mediterranean, Europe, South-East Africa, and the Western Pacific.

Therefore, we used correlation to highlight the link between hospitals, laboratories, inpatient facilities, and outpatient facilities and regression analysis to determine if the number of hospitals is determined by the number of clinical laboratories.

In the case of a connection between the behavior of some variables, regression analysis can be performed [45] in order to describe a phenomenon as the result of the action of one or more factors [46]. Thus, we can study and measure the dependency between two or more variables [47], and the regression model can be written as follows:

(1) $$\mathrm{Hospitals}=\beta_{0}+\beta_{1}\cdot \mathrm{Clinical}\_\mathrm{laboratories}+\beta_{2}\cdot \mathrm{inpatient}\_\mathrm{facilities}+\beta_{3}\cdot \mathrm{outpatient}\_\mathrm{facilities}$$

The data in this study are processed using software Eviews 9.5 and Excel and Vos programs.

## 4. A Bibliometric Analysis on the Lean Six Sigma Methods

Therefore, in order to analyze the most relevant concepts in the field, we used bibliometric analysis, the principal source of scientific articles analyzing Lean Six Sigma methods being the academic platform Web of Science and Pub Med.

First, we explored the content of all (1661) research articles related to the Lean Six Sigma method on the Web of Science in order to highlight the structure of the scientific field using content analysis, which inspects the most common words and the relationship between words.

In analyzing the network of co-occurrences, co-occurrences with a frequency of at least 20 times were taken into account with a correlation degree greater than 0.5. The analysis was done using the Vos program.

Exploring the valuable information provided by the word clouds, we tried to respond to the following main research question: What are the most common words found in the full scientific articles?

**Figure 1:**
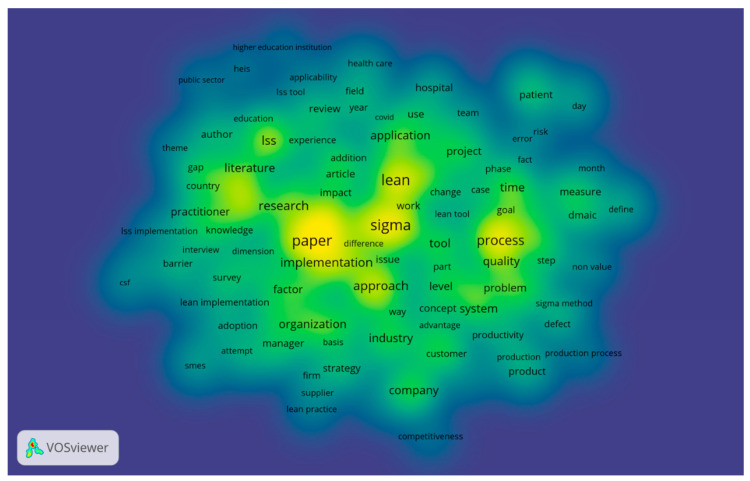
Most common words in scientific publications.

The empirical analysis proved that the most common words in the full content of the selected articles, apart from the keywords used, are: “process,” “sigma,” “lean,” “approach,” ”research,” “practitioner,” “time,” “quality,” “problem,” “industry,” “company,” “tool,” ”factor,” “organization,” “patient,” and ”hospital, (Figure 1), confirming Hypothesis 1.

**Figure 2:**
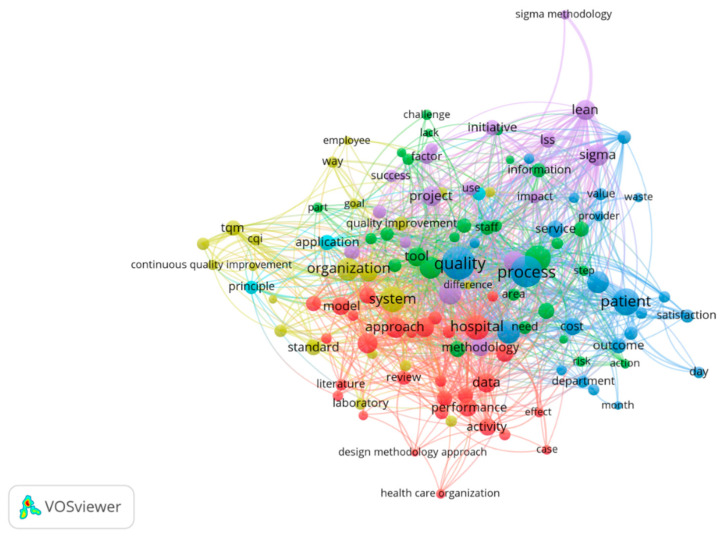
Word network in medical scientific publication content.

In order to analyze which words have a co-occurrence rate of at least 20 times frequency in the medical field, we used the first 720 abstracts on PubMed according to the number of citations, the empirical results highlighting the fact that the most common combinations in the most relevant studies in the field are: patient–cost–satisfaction–quality–outcome–process, lean–sigma–information–initiative–impact, tqm–cqi–organization–standard, and performance–data–activity–hospital (Figure 2). In order to highlight the combinations of words the most encountered, the most correlated words within the selection of articles were explored using the value of 0.5 as the threshold.

## 5. Empirical Results

Around the world, clinical laboratories are spearheading novel approaches to patient care and healthcare systems management. By leveraging their unique data and expertise, they are exploring new frontiers for the clinical laboratory profession in an era of rapid technological change.

**Figure 3:**
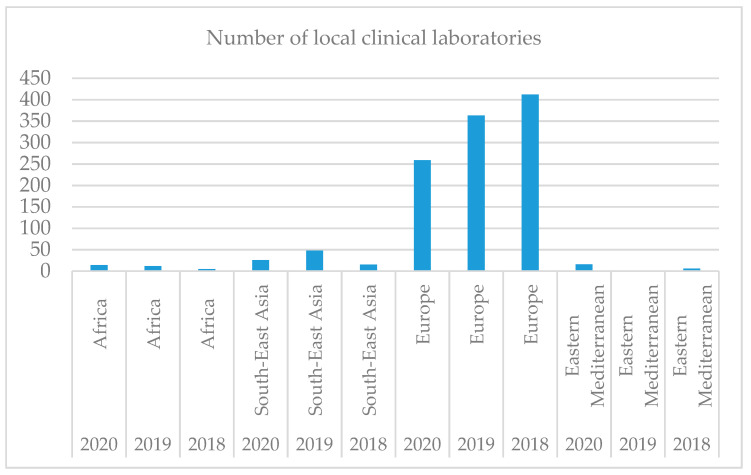
Number of clinical laboratories all over the world in 2018–2020.

As shown in Figure 3, clinical laboratories are not evenly distributed worldwide, the largest number being registered in Europe. With a large aging population, a shortage of healthcare workers, and the rising burden of chronic diseases, healthcare systems in the region cannot rely on lessons from Western Europe or the US. Solutions designed for this part of the world must address unique challenges and local needs. In Singapore, for example, the country is pushing ahead with a National Electronic Health Record (NEHR), which aims to provide a consolidated view of every patient’s health record across every interaction with the Singapore health system—including lab tests and results—from birth to death.

Over time, the number of clinical laboratories decreases in all regions except Europe, registered as an ascendant trend. This fact can be explained by the awareness of the population and health personnel regarding clinical laboratories, which aids physicians in carrying out the diagnosis, treatment, and management of patients. Therefore, Hypothesis 2 is confirmed, Europe being the leader regarding the number of clinical laboratories.

**Figure 4:**
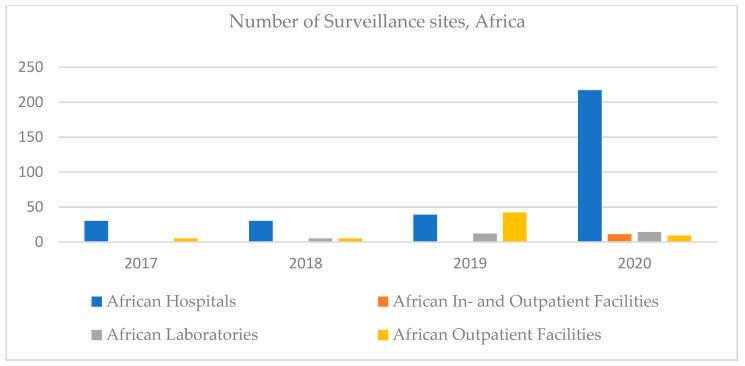
Number of surveillance sites, Africa, 2017–2020.

Referring to Africa, in 2017 there were no declared clinical laboratories. Over time, this number has grown slightly, but little compared to the number of hospitals. This can be a particularity of the African health system, with an emphasis on hospitals. The number of outpatient facilities increased until 2019, decreasing dramatically in 2020 when inpatient facilities were also registered (Figure 4).

**Figure 5:**
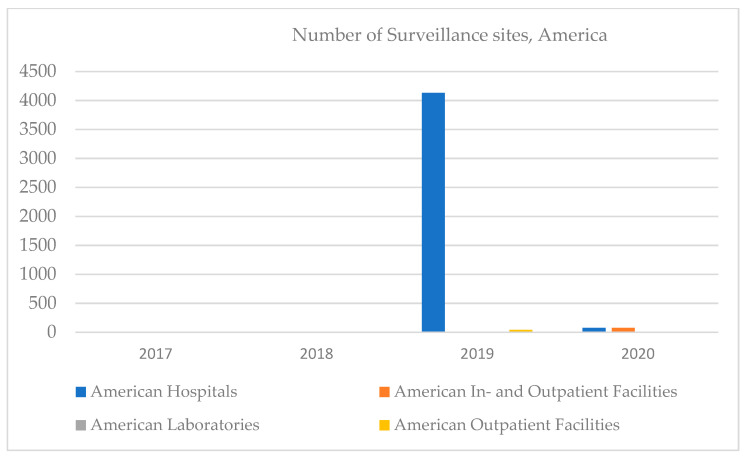
Number of surveillance sites, America, 2017–2020.

In America, in 2019, there were dramatic increases in the number of hospitals but decreases regarding the number of inpatient facilities. No clinical laboratories were reported in the 2017–2020 period; however, outpatient facilities started appearing in 2020 (Figure 5). Most of the major projects in the Clinical Lab 2.0 community are currently happening in the US, where pressure to reduce healthcare costs has reached astronomical proportions. While some Asian countries—such as Japan and Singapore—outperform the US on many key measures of healthcare systems efficiencies, most of the region faces similar pressures associated with rising costs and insufficient outcomes. 

**Figure 6:**
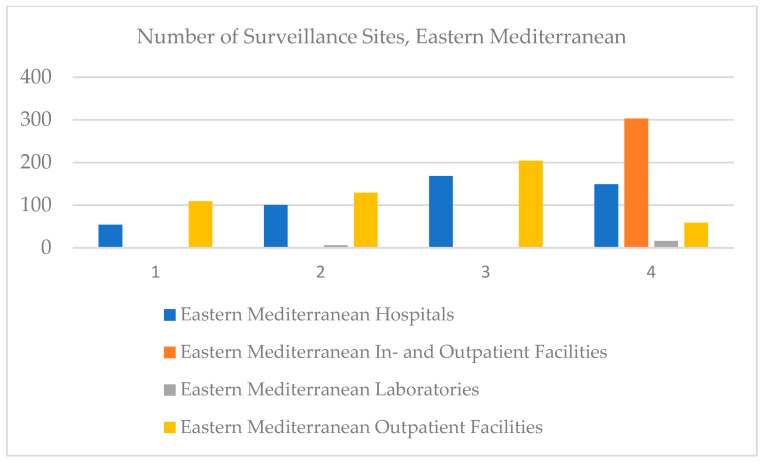
Number of surveillance sites, Eastern Mediterranean, 2017–2020.

In the Eastern Mediterranean, clinical laboratories were registered in 2018, and in 2020, an increase was registered. Paradoxically, the number of hospitals increased until 2019, but in 2020 it decreased, especially since in 2020, the whole world faced the health crisis caused by COVID-19. The same trend was registered by outpatient facilities, and values regarding in- and outpatient facilities were not recorded (Figure 6).

**Figure 7:**
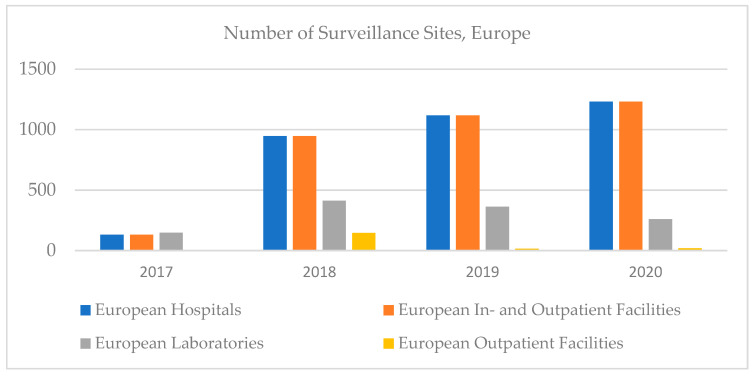
Number of surveillance sites, Europe, 2017–2020.

In Europe, the number of hospitals, outpatient facilities, and in- and outpatients increased until 2020, then decreased, and the number of clinical laboratories decreased; however, the highest increase in clinical trials was recorded in Europe (Figure 7).

**Figure 8:**
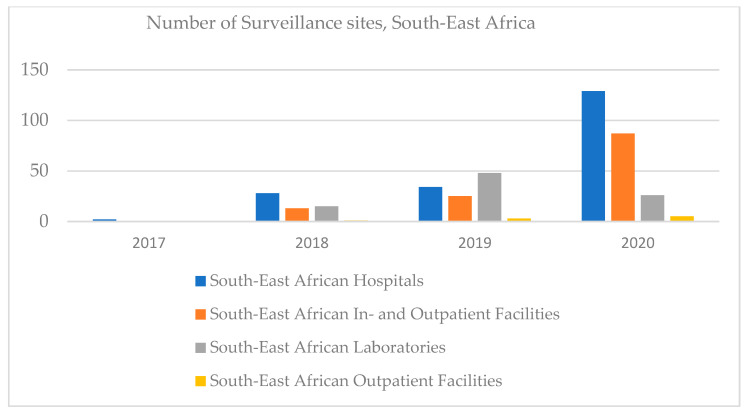
Number of surveillance sites, South-East Africa, 2017–2020.

In South-East Africa, the number of hospitals and in- and outpatient facilities increased, outpatient facilities being registered in 2020, and clinical laboratories increased until 2019, then decreased (Figure 8).

**Figure 9:**
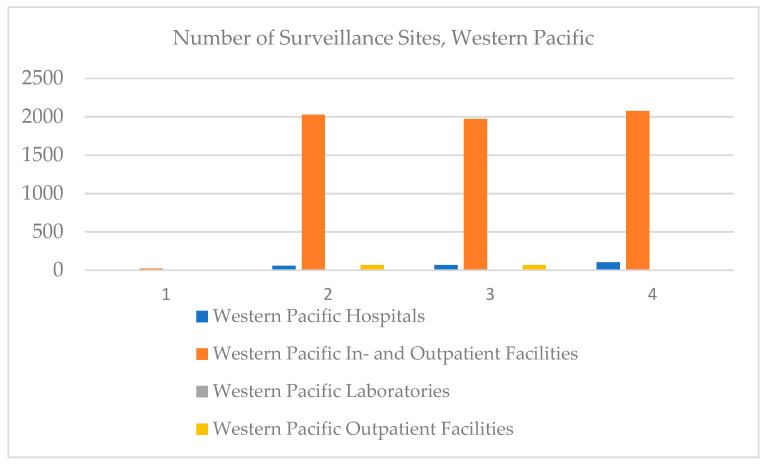
Number of surveillance sites, Western Pacific, 2017–2020.

In the Western Pacific, the number of in- and outpatient hospitals has increased over time, the number of outpatient facilities has stagnated, and there are no on-board clinics registered (Figure 9).

In Africa, the hospitals variable is strongly and directly correlated to clinical laboratories and inversely and weakly correlated to outpatient facilities. Clinical laboratories are medium and direct correlated to clinical laboratories (Table 2).

In America, the hospitals variable is strongly but inversely correlated to outpatient facilities (Table 3).

In the Eastern Mediterranean, the hospitals variable is weak and directly correlated to outpatient facilities and very weak and inversely correlated to clinical laboratories, the number of clinical laboratories being very strong and inversely correlated to outpatient facilities (Table 4).

In Europe, the hospitals variable is very strong and directly correlated to outpatient facilities and medium and directly correlated to the number of laboratories. The number of laboratories is strong and directly correlated to outpatient facilities (Table 5).

In South-East Africa, the hospitals variable is strong and directly correlated to outpatient facilities, almost perfectly and directly correlated to in- and outpatient facilities, and weak and inversely correlated to clinical laboratories. In- and outpatient facilities are very weak and inversely correlated to clinical laboratories and very strong and directly correlated to outpatient facilities. The number of clinical laboratories is medium and directly correlated to outpatient facilities (Table 6).

In the Western Pacific, the hospitals variable is strong and directly correlated to in- and outpatient facilities and very strong but inversely correlated to outpatient facilities. In- and outpatient facilities are strong and inversely correlated to outpatient facilities (Table 7).

Each region has its own specificities, but by referring to the entire population, we can deduce that the number of hospitals is very strong and directly correlated to the number of clinical laboratories, medium correlated and directly correlated to outpatient facilities, and very weak and inversely correlated to in- and outpatient facilities. The number of laboratories is very weak and directly correlated to outpatient facilities. The number of in- and outpatient facilities is very weak and directly correlated to clinical laboratories and outpatient facilities (Table 8), confirming Hypothesis 3.

In order to identify the dependency between hospitals and the number of clinical laboratories, in- and outpatient facilities, and outpatient facilities, we realized a regression model, where the number of hospitals represents the dependent variable and the independent variables are in- and outpatient facilities, clinical laboratories, and outpatient facilities. As it can be seen in Table 9, the link between the number of hospitals and in- and outpatient facilities, clinical laboratories, and outpatient facilities is very strong, the number of hospitals variation being explained by the cumulative variation of the independent variables (Table 9).

The model is valid, with a probability of 95% (Table 10) and only the variable clinical laboratories being statistically significant (Table 11).

Therefore, when the number of clinical laboratories increases by one unit, the number of hospitals also increases by 0.7 units (the link being direct). Thus, increasing the number of clinical laboratories will increase the number of hospitals, therefore increasing population health, confirming Hypothesis 4 (Table 11).

Efficiency has emerged as a central goal to the operation of health care organizations. By increasing the number of clinical laboratories, implementing Lean Six Sigma will increase their efficiency and the cost will be reduced, overall increasing the healthcare system efficiency and leading to a better status of health.

Although achieving organizational efficiency is necessary for healthcare organizations, given the changes that are currently occurring all over the world in the healthcare system, it is important for healthcare managers to maintain a certain level of slack in order to respond to environmental demands and have the resources needed to improve their performance.

## 6. Discussion

Despite a plethora of research, Lean thinking in healthcare settings is still in its infancy and often lacks the crucial building blocks required for success [48]. 

To achieve benefits in terms of quality and costs, Six Sigma started being implemented in several clinical laboratories around the world. A lack of Lean externalities research in medical laboratories suggests the need for an extension of our study scope. To date, there have been a limited number of reviews of Lean or Lean interventions in healthcare [49], and much less if we discuss laboratories.

Having studied Lean Six Sigma methodology in a clinical laboratory environment and the taken steps, effects, challenges, and specifically used instruments presented in Web of Science articles in the last 4 years, we identified some major aspects related to project management. 

Projects are all individual and each of them present different challenges and effects; however, there are some main keys to ensure the LSS project success.

An important focus is placed on the training side. Projects usually ensure training at the beginning of the project, but, as mentioned by some of the authors, it should represent a continuous process. Moreover, during training, people should be assured that the LSS project brings improvements and advantages to the company or laboratory, without threatening their jobs, highlighting the importance of their work for the overall process, as well as encouraging their feedback.

People’s perceptions of the project play an important role in their involvement, openness, and motivation. Additionally, they are the ones who contribute to establishing a continuous improvement culture, which is a desirable state for long-term results. 

By doing the listed actions, some major presented challenges are avoided:

(1) Conflicts, because people understand they are all operating for the same objectives, they are part of the same project, and they become aware of their job implications.
(2) Resistance to change, as they are no longer frightened about losing their jobs as a result of optimization.
(3) Giving up the project in the short term.

A lack of research was found in the Lean Six Sigma implementation in the health laboratory field on a medium and long-term basis. In our future research, we are interested in this aspect, as well as in an analysis of LSS challenges in clinical laboratories, to identify an appropriate systemic approach to overpass them and ensure successful LSS application. 

## 7. Conclusions

Important in the testing services in medical laboratories is the creation of a flexible balance between quality–response time and minimizing the cost of the service.

The Lean method applied in laboratories, associated with process digitization, allows for:

- The saving of time between the moment of collecting the samples and the transmission of the test results, with the interpretation of the specialist doctor, assisted by AI
- A reduction in the cases of diagnostic errors, development of cooperation with AI-database query (permanently updated), selection of similar/comparable cases, comparative analysis, optimization of AI decision (maximum 2–3 alternatives), and final decision by comparison with the interpretation of the specialist.

Beyond the different Lean methods implemented so far in the medical sector, each company can adapt the model according to its needs and each company has its own specifics and its own organizational culture, and Lean implementation will have a unique approach.

Considering the specific character of Lean implementation, the specialized literature mainly presented case studies, which highlighted the different aspects related to the duration and quality, and even less, a comparative analysis on implementation results. Recent studies focus on the synthesis of value-added elements from published case studies. Santos et al. [50] provide an overview of the key factors that can promote lean implementation based on Web indexed articles in 2009–2018 and by investigating and comparing how conceptual and analytical articles address tools/methods, application fields, implementation barriers and facilitators, and positive and negative impacts. 

Moreover, beyond the effects on business efficiency health services, recent concerns [49] identify the role of value stream mapping in healthcare services advocating for more standardization and the broadening of impact measurement indicators from predominantly operational indicators to social sustainability indicators. However, the limits of standardization stop at the specifics of the technological flow and the content of the medical services offered, there being significant differences between the activity of testing laboratories, lamella processing, etc., and other services such as family medicine. An analysis of the literature focused on the application of Lean in such laboratories highlighted a few articles published in WoS in recent years. It is also the reason why in our work, we carried out specific bibliometric research and systematized the results. 

Even though Lean’s results seem promising, so far, the findings do not allow for a final conclusion to be drawn on their impact or positive challenges when introduced into the healthcare sector [18], especially on a long-term basis.

The main goal was to identify both externalities–positive and negative–(multidimensional in the implementation of Lean, from streamlining processes and reducing processing time to employee perception and customer satisfaction) and highlighting the flexibility of organizing activity and concerns for the future, for example, to analyze the degree of digitization and the dynamics of processing technologies, considering that digitization is an opportunity for quality, productivity, and updating of processing technologies.

The results obtained identified:

- Lean approaches are evolving to better meet the needs of healthcare [51,52,53];
- Emphasis on the presentation of case studies on the subdomains of medical services and less systematization of results or through Lean post-implementation impact analyzes, on the medium and long term [54,55,56];
- Highlighting the benefits and less negative externalities, although many studies mention the reluctance of employees [57,58,59];
- The benefits are strictly analyzed on the phases of service provision (in our research, the testing and interpretation of the result) without highlighting the impact on the value chain of patient treatment—from reducing diagnostic errors to reducing patient waiting time and cooperation between medical specializations for optimizing interventions and treatment schemes [60,61,62].

From the research identified in recent years and published in WoS, we find the quasi-lack of concern for the analysis of the benefits of integrating databases with previous processing in the form of real-time operational big data to complement human expertise in interpreting results and defining diagnosis. It cannot fully take over the service of analysis and interpretation of analyses (slides, etc.), human involvement being, in our opinion, strictly necessary, but it can help substantially in the iteration and selection of similar situations. Therefore, the digitalization of histopathological medical testing laboratories can be associated with the implementation of Lean, as a tool for optimizing the medical service as a whole, from the perspective of medical act quality and professional satisfaction of laboratory experts and business efficiency, including managerial optimization.

A comparative analysis of the content of the published articles regarding Lean implementation in medical analysis laboratories, with emphasis on histopathological testing laboratories, highlighted the following:

- Lean, in its various variants, is a managerial method for streamlining business and optimizing technological flow, which can be recommended in the process of technological transfer and digitization of the activity [63,64,65,66,67];
- Although the employees of the laboratories were reluctant to apply the method for fear of dismissal, it is obvious that in such activities, we witness not job destruction but definitely a job content enrichment process, which means digital skills and permanent updating of professional knowledge [68,69,70];
- The quality of the results is consolidated and the risk of erroneous diagnosis is reduced, all the more so as complementary methods of validation of the final results are adopted, such as querying databases for comparison with similar situations [67,68];
- The role of human resources in the final decision remains essential, but the lean method and digitization allow flexibility in the stages of analysis and focus on particular situations new or more difficult to diagnose and interpret the results, on average reducing service life, with significant benefits to the patient and of the integral treatment process [70,71,72,73];
- Teamwork and the association of specific complementary expertise, including remote communication, remains the future solution for the quality of medical diagnostic service in laboratories [74,75,76];
- The professional competence of laboratory medicine experts is associated with the need for technological skills (for the use of high-performance equipment) and software skills, including digital software. The model and structure of employment in laboratories changes and leaves room for mixed teams, from the perspective of age groups, the lack of experience of young graduates being solved in two ways: the traditional, collaboration with seniors, but also by accepting AI as a “dialogue partner” in querying constantly updated historical databases. Thus, the risk of burnout during peak periods in testing disappears or decreases significantly, productivity increases on average, and the economy of operating costs can be redistributed to investments in technology and incentives through wages [77,78,79];
- What is certain is that the implementation of Lean is currently done on another level, pursuing multiple objectives, including indicators of social impacts (employees and customers) and ecological impacts (the processes of processing, storage, and waste management, along with those specific to medical service quality), and the reduction of diagnostic errors [80,81];
- Conceptually, the Lean model is transformed from the mechanistic approach of optimizing the manufacturing phases—as a strict objective of business efficiency—to the dual approach, that of a technological and financial optimum associated with social, societal, and environmental impacts: a) accessibility, quality, and patient satisfaction, b) green activities, and c) continuous professional development, change of employment model, and work content, respectively [82,83].

The purpose of the research is confirmed by the analyses performed:

- Identifying the concerns of specialists and the laboratory medical services sector in optimizing medical services by implementing the Lean method in its various variants
- Comparative analysis of the implemented models, with emphasis on measuring externalities and delimiting trends in reforming/modernizing the method, to include a comprehensive approach to the impact of Lean method implementation
- Analysis of available databases and identification of deficit and information asymmetry

As novelty elements, we can mention: the realization of a bibliometric analysis of content focused on the application of Lean in medical laboratories, a critical state analysis of the existing statistical information and recommendations, and the delimitation of the coordinates of the laboratory testing medical services mode, organized according to the Lean digitized principles, with the incorporation of AI as a “working” tool.

Lean Six Sigma has also managerial implications as it represents a management approach for driving innovating processes in order to achieve better results. It implies processes efficiency, aiming for innovation and growth on long-term processes registering gradual and continuous improvement. Thus, it is conducive to attaining superior financial performance by addressing new needs, differentiating products and services, or adjusting business lines to new processes [84,85]. LSS tools are associated with project scope, program phase, and functional area and project outputs in program management organizations, improving the quality, speed, and financial benefits of the managed programs [86].

The limitations of the article are reflected by the missing information regarding clinical laboratories implementing LSS. The analysis is restricted, also to searching the Web of Science and PubMed online databases. Thus, extension to other research databases would help achieve a clearer overview. A further classification of the clinical laboratories based on their activity type would deepen the research.

## Figures and Tables

**Table 1:** WoS papers focused on LSS implementation in medical laboratories.

No.	Authors. Article Title. Year [Reference]	Area. Place	Method. Mentioned Instruments	Positive Effects	Negative Effects
1	Durur, F., & Akbulut, Y. Lean Methodology for Pathology Laboratories: A Case Study from a Public Hospital. 2019 [23]	Pathology laboratory of a public hospital. Ankara, Turkey	A five-phase plan: the support of senior management, observation, training of employees, VSM drawing, and VSM map creation. VSM, fishbone diagram, and Pareto analysis, 5S	Decrease of waiting time and increase of sample number	Lean management philosophy takes a long time to adopt
2	Isack, H.D., Mutingi, M., Kandjeke, H., Vashishth, A., et al. Exploring the adoption of Lean principles in medical laboratory industry Empirical evidences from Namibia. 2018 [24]	Medical laboratories. Namibia	A seven-step implementation strategy is proposed. Standard operating procedures, root cause analysis, overall equipment effectiveness, visual management, PDCA (Plan-Do-Check-Act) cycle, inventory control cards, Fishbone diagram, 5 Whys, Pareto analysis	Quality improvement, operational performance, turnaround time, customer retention/satisfaction, market share, employee motivation, cost reduction, and reduced waste. The main enablers are top management involvement, adequate training, and proper planning.	Major barriers are a lack of support from management, financial constraints, and staff resistance to change.
3	Letelier, P., Guzmán N., Medina G., et al. Workflow optimization in a clinical laboratory using lean management principles in the pre-analytical phase. 2020 [25]	Clinical laboratory. Temuco, Chile	Not mentioned. By deduction: 1. Pre-intervention Lean training 2. Intervention and data collection and analysis 3. New workflow, Workflow analysis, and Kaizen event	A reduction in turnaround times, improvement in the delivery time of test results, optimizing teamwork and assertive communication.	In the analytical and post-analytical phases (not intervened), an increase in turnaround times was observed in some cases.
4	Kovach, J.V., & Ingle, D. Using Lean Six Sigma to Reduce Patient Cycle Time in a Nonprofit Community Clinic. Quality Management in Health Care. 2019 [26]	Nonprofit healthcare clinic. Texas	Lean-oriented process analysis tools/methods within Lean Six Sigma’s DMAIC, brainstorming, interview, swim-lane diagram/cross-functional flowchart, process map, SIPOC, FMEA, training, and visual management tools. A nine-step plan is described in the article.	Reducing patient cycle time by 22% and increasing patient visit capacity, reduction of patient wait time, increased appointment availability, increased quality of patient care, and increased clinic’s patient visits by 27%.	Quick exhaustion of resourcefulness in previous similar projects.
5	Moran, M.E., Sedorovich, A., Kish, J., Gothard, A., et al. Addressing Behavioral Health Concerns in Trauma: Using Lean Six Sigma to Implement a Depression Screening Protocol in a Level I Trauma Center. 2020 [27]	Trauma center. Midwest United States	LSS DMADV. Project charter, MGP (Multigenerational Plan), VOC CTQ, focus group, questionnaire	PHQ-2 document location compliance increased from 60.74% to 80.56% and enhanced focus on patient behavioral health concerns, which resulted in a significant increase in CLP interventions.	Risks of biases specific to the region and institution as a whole, which may limit the generalizability.
6	Ahmed, S., Abd Manaf, N.H., & Islam, R. Effects of Six Sigma initiatives in Malaysian private hospitals. 2018 [28]	Eight private hospitals. Malaysia	Questionnaire study on Six Sigma effects	Six Sigma methodology binds all operational activities together and links the strategic and the operational level in a private hospital. Improves quality and business performance.	Hospital management should invest time to understand Six Sigma initiatives and incorporate them into management oversight and strategic planning for the continuous improvement of quality performance.
7	Ahmed, S., Abd Manaf, N.H., & Islam, R. Effect of Lean Six Sigma on Quality Performance in Malaysian Hospitals. 2018 [29]	15 hospitals. Malaysia	Questionnaire study. Control chart, histogram, Pareto chart, scatter diagram, PDCA, root cause analysis (RCA), balanced scorecard, benchmarking, 5 S, and five whys.	LSS and workforce management have a significant impact on the quality performance of Malaysian hospitals, whereas senior management commitment was found to have an insignificant relationship with quality performance.	Proper planning, prioritization, resource allocation, budgeting, training, and proper review and reward mechanisms are required as well as informal strategies to enhance relationships.
8	Persis, D.J., S., A., Sunder, M.V., G., R. Sreedharan, V.R., & Saikouk, T. Improving patient care at a multi-speciality hospital using Lean Six Sigma. 2020 [30]	Multi-speciality hospital. India	SDMMAICS (Select–Define–MeasureMap–Analyze–Improve–Control–Sustain), interview questionnaire, force field analysis, project charter, I-chart, VSM, process flowchart, Fishbone diagram, Pareto chart, and cost–benefit analysis, brainstorming, and virtual queuing model	Removed bottlenecks and improved key performance metrics: patient turnaround time (TAT), workforce utilization, and organizational profits, reduced staff and teams’ idle time with an annual saving of USD 44,000.	Criticisms and drawbacks review presented in the article.
9	Geerlinks, A.V., Digout, C., Bernstein, M., Chan, A., MacPhee, S., Pambrun, C., et al. Improving Time to Antibiotics for Pediatric Oncology Patients With Fever and Suspected Neutropenia by Applying Lean Principles. 2018 [31]	Pediatric Oncology. Tertiary care pediatric hospital. NovaScotia, Canada	DMAIC. VSM	Quality indicator: improve Time-to-Antibiotics (TTA) for children with chemotherapy-induced febrile neutropenia (from median of 99 to 59 min). Patients treated within 60 min improved from 12% to 47%, and those treated within 90 min from 39% to 74%.	Not mentioned
10	Randell, E.W., Short, G., Lee, N., Beresford, A., Spencer, M., Kennell, M., et al. Autoverification process improvement by Six Sigma approach: Clinical chemistry & immunoassay, 2018 [32]	Clinical chemistry and immunoassay. Three clinical laboratories. Newfoundland and Labrador, Canada	DMAIC	Over 890% increase in overall test and sample auto verification, improved turnaround time, and reduced time for manual verification. Mean weekly time MLTs spent reviewing results decreased from 16,785 ± 5461 (*n* = 10 weeks) before to 5439 ± 1573 s (*n* = 7 weeks)	Manual review time increased from 7.1 ± 3.9 s to 21.0 ± 7.1 s
12	De Oliveira, K.B., dos Santos, E.F., & Junior, L.V.G. Lean Healthcare as a Tool for Improvement: A Case Study in a Clinical Laboratory, 2017 [33]	Clinical Pathology and Pathological Anatomy, SaoPaulo State, Brasil	Process understanding, VSM, Design an Improvement plan	Future increase in added value in a future scenario to their services, reducing or eliminating waste in processes and increasing customer satisfaction, mainly by reducing waiting time.	Not mentioned

**Table 2:** Correlation matrix, Africa.

	Hospitals	In- and Outpatient Facilities	Laboratories	Outpatient Facilities
Hospitals	1			
In- and Outpatient Facilities		1		
Laboratories	0.744		1	
Outpatient Facilities	−0.187 *		0.489	1

* Correlation is significant at the 0.1 level (2-tailed).

**Table 3:** Correlation matrix, America.

	Hospitals	In- and Outpatient Facilities	Laboratories	Outpatient Facilities
Hospitals	1			
In- and Outpatient facilities		1		
Laboratories			1	
Outpatient Facilities	−0.99 ***			1

*** Correlation is significant at the 0.01 level (2-tailed).

**Table 4:** Correlation matrix, Eastern Mediterranean.

	Hospitals	In- and Outpatient Facilities	Laboratories	Outpatient Facilities
Hospitals	1			
In- and Outpatient facilities		1		
Laboratories	−0.084		1	
Outpatient Facilities	0.309		−0.978 ***	1

*** Correlation is significant at the 0.01 level (2-tailed).

**Table 5:** Correlation matrix, Europe.

	Hospitals	In- and Outpatient Facilities	Laboratories	Outpatient Facilities
Hospitals	1			
In- and outpatient facilities	1	1		
Laboratories	0.567	0.695	1	
Outpatient facilities	0.977	0.200	0.702 ***	1

*** Correlation is significant at the 0.01 level (2-tailed).

**Table 6:** Correlation matrix, South-East Africa.

	Hospitals	In- and Outpatient Facilities	Laboratories	Outpatient Facilities
Hospitals	1			
In- and Outpatient facilities	0.995 *	1		
Laboratories	−0.137	−0.038	1	
Outpatient facilities	0.891 **	0.932	0.327	1

*, ** Correlation is significant at the 0.1 level (2-tailed) and at the 0.05 level (2-tailed).

**Table 7:** Correlation matrix, Western Pacific.

	Hospitals	In- and Outpatient Facilities	Laboratories	Outpatient Facilities
Hospitals	1			
In- and Outpatient facilities	0.885 *	1		
Laboratories			1	
Outpatient Facilities	−0.983	−0.854		1

* Correlation is significant at the 0.1 level (2-tailed).

**Table 8:** Correlation matrix, all over the world.

	Hospitals	In- and Outpatient Facilities	Laboratories	Outpatient Facilities
Hospitals	1			
In- and Outpatient facilities	−0.063	1		
Laboratories	0.931	−0.073 *	1	
Outpatient Facilities	0.372	−0.003	0.058 *	1

* Correlation is significant at the 0.1 level (2-tailed).

**Table 9:** Regression Statistics.

Multiple R	0.984075
R Square	0.968403
Adjusted R Square	0.921008
Standard Error	101.1219
Observations	6

**Table 10:** ANOVA.

	df	SS	MS	F	Significance F
Regression	3	626,809.8	208,936.6	20.43264	0.047019
Residual	2	20,451.26	10,225.63		
Total	5	647,261.1			

**Table 11:** Regression coefficients.

	Coefficients	Standard Error	t Stat	*p*-Value	Lower 95%	Upper 95%
Intercept	140.5385	65.38541	2.149386	0.164609	−140.792	421.8692
In- and Outpatient facilities	0.000786	0.018965	0.04146	0.970696	−0.08082	0.082388
Clinical Laboratories	0.697335	0.096426	7.231833	0.018589	0.282448	1.112221
Outpatient Facilities	0.636223	0.250949	2.535271	0.126682	−0.44352	1.715968
